# Evaluation of T-activated proteins as recall antigens to monitor Epstein–Barr virus and human cytomegalovirus-specific T cells in a clinical trial setting

**DOI:** 10.1186/s12967-020-02385-x

**Published:** 2020-06-17

**Authors:** Nina Körber, Uta Behrends, Ulrike Protzer, Tanja Bauer

**Affiliations:** 1 Institute of Virology, Helmholtz Zentrum München/Technical University of Munich, School of Medicine, Schneckenburgerstr. 8, 81675 Munich, Germany; 2 Children’s Hospital, School of Medicine, Technical University of Munich, Munich, Germany; 3 Research Unit Gene Vectors, Helmholtz Zentrum München, Munich, Germany; 4 German Center for Infection Research (DZIF), Partner Site, Munich, Germany

**Keywords:** EBV, HCMV, T cells, Recall antigens, Immune monitoring, Clinical trials

## Abstract

**Background:**

Pools of overlapping synthetic peptides are routinely used for ex vivo monitoring of antigen-specific T-cell responses. However, it is rather unlikely that these peptides match those resulting from naturally processed antigens. T-activated proteins have been described as immunogenic and more natural stimulants, since they have to pass through antigen processing and comprise activation of all clinically relevant effector cell populations.

**Methods:**

We performed comparative analysis of numbers and cytokine expression pattern of CD4 and CD8 T cells after stimulation with recombinant, urea-formulated T-activated EBV-BZLF1, -EBNA3A, and HCMV-IE1, and -pp65 proteins or corresponding overlapping peptide pools. Freshly isolated and cryopreserved PBMC of 30 EBV- and 19 HCMV-seropositive and seven EBV- and HCMV-seronegative subjects were stimulated ex vivo and analysed for IFN-γ, TNF and IL-2 production by flow cytometry-based intracellular cytokine staining.

**Results:**

T-activated proteins showed a high specificity of 100% (EBV-BZLF1, HCMV-IE1, and -pp65) and 86% (EBV-EBNA3A), and a high T-cell stimulatory capacity of 73–95% and 67–95% using freshly isolated and cryopreserved PBMC, respectively. The overall CD4 T-cell response rates in both cohorts were comparable after stimulation with either T-activated protein or peptide pools with the exception of lower numbers of CD8 T cells detected after stimulation with T-activated EBV-EBNA3A- (*p* = 0.038) and HCMV-pp65- (*p* = 0.0006). Overall, the number of detectable antigen-specific T cells varied strongly between individuals. Cytokine expression patterns in response to T-activated protein and peptide pool-based stimulation were similar for CD4, but significantly different for CD8 T-cell responses.

**Conclusion:**

EBV and HCMV-derived T-activated proteins represent innovative, highly specific recall antigens suitable for use in immunological endpoint assays to evaluate success or failure in immunotherapy clinical trials (e.g. to assess the risk of EBV and/or HCMV reactivation after allogenic hematopoietic stem cell transplantation). T-activated proteins could be of particular importance, if an impaired antigen processing (e.g. in a post-transplant setting) must be taken into account.

## Background

Epstein–Barr virus (EBV) and human cytomegalovirus (HCMV) are ubiquitous human herpesviruses, which establish persistent infections in the human population, while maintaining the capacity for reactivation to lytic infection [1–3]. Primary infection with EBV and HCMV is in most of the cases asymptomatic, but some individuals develop infectious mononucleosis (IM) (EBV) [4, 5] or a mononucleosis-like illness (HCMV) [6]. Both, EBV and HCMV can cause severe disease in immunocompromised individuals in whom the protective antiviral T-cell response is diminished [1], e.g. 80% of posttransplant lymphoproliferative disorders are associated with EBV infection [7]. EBV infection is also implicated in the pathogenesis of several life-threatening or disabling malignant and non-malignant human diseases [8], like Burkitt and Hodgkin lymphoma [9], nasopharyngeal carcinoma [10, 11], as well as autoimmune disorders such as multiple sclerosis [12]. Regarding HCMV, a sufficient risk stratification for solid organ transplantation is crucial to find the adequate dosage of immunosuppression to prevent recurrent HCMV reactivations on the one hand, but to avoid graft rejection on the other hand [13–15].

Hence, highly sensitive and validated immune monitoring assays are needed to assess the EBV- and HCMV-specific cell-mediated immunity (CMI) in e.g. transplant patients or as an immunological endpoint in immunotherapy trials to monitor secondary or exploratory immune response endpoints (i.e. to measure vaccine immunogenicity or summarize several longitudinal immunogenicity responses) and to allow for therapy decisions and correlation of assay results with clinical outcome. Immunoassays suitable to determine the frequency of antigen-specific CD4 and CD8 T cells (e.g. enzyme-linked immunosorbent assay (ELISA), enzyme-linked immunospot (ELISpot)- and flow cytometry-based assays) require an antigenic component ideally able to (i) reflect the in vivo situation including interaction of antigen presenting cells (APC) and effector cells, (ii) ensure highly sensitive monitoring while maintaining a high specificity, (iii) induce major histocompatibility complex (MHC) class I and II restricted T-cell responses, (iv) induce different effector functions, and (v) including all clinically relevant immune cells and their dynamic interaction.

The choice of immunogenic stimulants for virus-specific CD8 T-cell monitoring is hampered by the fact that CD8 T-cell reactivation requires antigen processing via the natural endogenous class I presentation pathway [16]. Exogenous full-length (viral) proteins and virus lysates (e.g. HCMV lysate) are usually processed by APC via the exogenous antigen processing pathway and presented by MHC class II molecules, and therefore they can only be used for CD4 T-cell monitoring [16]. In contrast, soluble peptides with an appropriate length of amino acids (aa), can directly bind to MHC class I molecules located on the surface of cells without prior antigen processing [16]. Therefore, most immune monitoring approaches are based on the use of overlapping synthetic peptide pools (PP), spanning the entire aa sequence of the desired viral protein. However, functionality of APC to capture and process viral antigens for MHC class II and I restricted epitope presentation to viral-specific T cells is not considered in this approach [16]. Pools of overlapping peptides have been used for epitope mapping [17, 18], investigating T-cell responses in vaccine development [19–21], and immunotherapies [22, 23]. The use of PP, however, has several drawbacks: commonly used PP contain 15–20 mers, which is at least for MHC class I restricted epitopes a suboptimal length [24, 25]. Additionally, aa length of the single peptides as well as the position of the T-cell epitope within a peptide can greatly influence the assay sensitivity [26]. The high number of peptides within one pool may also affect the outcome of T-cell monitoring as it is not known to what extent individual peptides interfere with each other in large pools of > 100 peptides [27].

As synthetic peptides can directly bind to MHC molecules without requiring antigen processing, impaired antigen processing and presentation, which may appear in certain patient cohorts is not taken into account. This is extremely important when dealing with immunocompromised patients (e.g. transplant recipients), because it is well known that immunosuppressive drugs interfere with maturation and functionality of APC [28–31]. PP-based immune monitoring in such patient cohorts may result in an overestimation of responding T cells in vitro, probably not reflecting the in vivo situation in patients [32]. These facts suggest that synthetic PP probably do not reflect the naturally processed peptide repertoire and may be a sub-optimal antigenic component for antiviral immune monitoring approaches of certain patient cohorts.

Specifically engineered recombinant EBV- and HCMV-derived T-activated proteins (TP) are protein antigens that have been urea-formulated (T-activation technology, Lophius Biosciences, Regensburg, Germany) to enter the endogenous antigen presentation pathway more efficiently [33]. Thus, TP-derived peptides are not only presented by MHC class II, but also due to cross-presentation by MHC class I molecules [33]. Due to their natural antigen processing, TP overcome some limitation of PP-based immune monitoring and by activating all clinically relevant immune cell populations they provide an innovative antigenic component for different kinds of immunoassays [33].

Just recently, it was shown that HCMV-derived IE-1 and pp65 TP represent suitable stimulants to monitor functionality of HCMV-specific immunity [34–36]. However, a comprehensive comparative study of TP and PP induced CMI has not yet been performed.

The magnitude of virus-specific CD4 and CD8 T-cell responses and their diversity (i.e. the cytokine expression pattern) are considered significant parameters in evaluating the efficacy of an immune response [37–39]. In order to assess suitability and comparability of currently used immunogenic stimulants for immune monitoring of EBV- and HCMV-reactive T cells in clinical trial settings (e.g. to assess EBV- and/or HCMV-specific cell mediated immunity after solid organ or allogenic hematopoietic stem cell transplantation [40, 41]), we determined the number and cytokine expression pattern of CD4 and CD8 T cells upon stimulation with two EBV- and two HCMV-derived TP and their corresponding overlapping PP. Further, we determined the specificity of TP and the TP- and PP-induced response rates when using different specimen material [i.e. freshly isolated versus cryopreserved peripheral blood mononuclear cells (PBMC)].

Our study provides important insight into the validity of these new immunogenic virus-specific stimulants for endpoint monitoring in immunotherapy trials, confirming TP as an innovative tool for antiviral immune monitoring. Considering the natural antigen processing pathway is advantageous for immune monitoring of immunocompromised patient cohorts, where impaired antigen processing needs to be considered.

## Materials and methods

The authors acknowledge the concept of the Minimal Information About T-cell Assays (MIATA) framework [42, 43]. Therefore, detailed information is provided as structured in the five modules proposed by MIATA (miataproject.org/).

### The sample

#### Subjects

Peripheral blood was taken by venepuncture from 37 healthy donors (25 women, 12 men; mean age: 38 years) with the approval of the local ethic committee (School of Medicine, Technical University of Munich). Informed consent was obtained from all participating subjects prior to their inclusion. Donors were either HCMV- (n = 19) and/or EBV-seropositive (n = 30) or -seronegative (n = 7) as determined by pre-testing with a diagnostic assay (ELISA for EBV-/HCMV-serostatus and EBV-Immunoblot). Additional testing showed that none of the EBV-seropositive subjects had an EBV primary infection or reactivation status at the day of venepuncture, indicated by absence of detectable EBV VCA IgM.

#### Isolation and cryoconservation of PBMC

Within 4 h after collection of heparinized whole blood human PBMC were separated by Ficoll density gradient (human Pancoll, PAN-BIOTECH, Aidenbach, Germany) as described previously [44]. The median PBMC number obtained per mL whole blood was 1.95 × 10^6^ PBMC with a median viability of 96%. PBMC were either directly used for flow cytometry-based intracellular cytokine staining or frozen at 5 × 10^6^ PBMC per vial in 1.8 mL cryotubes (Thermo Scientific, Roskilde, Denmark) in a concentration of 1 × 10^7^ PBMC per 1 mL freezing medium (fetal calf serum (FCS) (Life Technologies, Darmstadt, Germany), supplemented with 10% DMSO (Sigma-Aldrich, Steinheim, Germany), using a freezing container (Mr. Frosty, Thermo Scientific, Roskilde, Denmark) and put on − 80 °C. After 24 h PBMC were stored in liquid nitrogen until further use.

#### Thawing and resting of PBMC

PBMC were thawed at 37 °C using RPMI1640 medium supplemented with 10% FCS and 1% penicillin–streptomycin (PenStrep, Life Technologies, Invitrogen, Darmstadt, Germany) (abbr.: RPMI-10) as described previously [44]. Cells were counted with an automated cell counter (Vi-cell XR, Beckman Coulter, Krefeld, Germany). The median cell recovery after thawing was 4.88 × 10^6^ PBMC per vial with a median viability of 94%. For a standard resting procedure PBMC were incubated for 18 h at 37 °C in a humidified atmosphere at 5% CO_2_ in a concentration of 2 × 10^6^ PBMC/mL RPMI-10. After resting the median cell recovery was 4.2 × 10^6^ PBMC per vial with a median viability of 95%.

### Flow cytometry-based intracellular cytokine staining

#### Stimulatory agents

The following stimulatory agents were used in this study: overlapping peptide pools of EBV-BZLF1 (PepMix™ EBV (BZLF1), product code: PM-EBV-BZLF1, 59 peptides), EBV-EBNA3A (PepMix™ EBV (EBNA3a), product code: PM-EBV-EBNA3a, 234 peptides), HCMV-IE1 (PepMix™ HCMVA (IE-1), product code: PM-IE1, 120 peptides), and HCMV-pp65 (PepMix™ HCMVA (pp65), product code: PM-PP65-1, 138 peptides) (JPT Peptide Technologies, Berlin, Germany), consisting of 15 mers overlapping by 11 aa; recombinant urea-formulated T-activated^***®***^ EBV-BZLF1, EBV-EBNA3A, HCMV-IE1, and HCMV-pp65 proteins (Lophius Biosciences, Regensburg, Germany). The optimal assay concentration of PP and TP was identified in previous titration experiments.

#### Ex vivo stimulation

1 × 10^6^ viable, freshly isolated or overnight rested PBMC were distributed in 150 µL RPMI-10 containing costimulatory antibodies to ensure effective T-cell stimulation (1 μg/mL anti-CD28; BD Biosciences, Heidelberg, Germany) in one well of a 96-well polypropylene U-bottom microtiter plate. Cells were stimulated with PP in a concentration of 1 µg/mL (EBV and HCMV PP). Stimulation with TP was performed with a concentration of 10 µg/mL (EBV-BZLF1), 15 µg/mL (EBV-EBNA3A), 3 µg/mL (HCMV-pp65), and 15.6 µg/mL (HCMV-IE1), respectively. A mock stimulated sample was run in parallel to define background activity. After 3 h of incubation at 37 °C in 5% CO_2_, 10 μg/mL of secretion blocker Brefeldin A (Sigma-Aldrich, Munich, Germany) was added to the cell suspension and incubation was carried out for additional 4 h at 37 °C in 5% CO_2_. After the re-stimulation period intracellular cytokine staining (ICS) was performed.

#### Intracellular cytokine staining

Following our standard operating procedure (SOP) for ICS, re-stimulated PBMC were labelled with the LIVE/DEAD^®^ Fixable Near-IR Dead Cell Stain Kit (Invitrogen, Darmstadt, Germany) for 30 min on ice in the dark and washed twice with 200 µL FACS buffer (BD Pharmingen Stain Buffer, BD Biosciences). Afterwards, PBMC were fixed and permeabilized for 20 min on ice in the dark using 100 µL/well BD Cytofix/Cytoperm Kit (BD Biosciences). After two wash steps with 200 µL/well Perm/Wash solution (BD Cytofix/Cytoperm Kit; BD Biosciences) PBMC were stained intracellularly with the antibodies listed in Additional file 1: Table S1 in a total volume of 80 µL Perm/Wash buffer for 30 min on ice in the dark. Cells were washed twice and finally re-suspended in 300 µL FACS buffer for acquisition. Cells were stored cold and in the dark until acquisition.

#### Data acquisition

Acquisition of samples was performed within 6 h after staining using a LSR2/LSR Fortessa flow cytometer equipped with a 96-well plate reader and FACSDiva Software V.6.0 (Becton–Dickinson, Heidelberg, Germany). Photomultiplier voltages were adjusted with the help of unstained cells for all parameters. Analysis was performed on at least 1.5 × 10^5^ living lymphocytes using the software FlowJo version 9.7 (Treestar, Ashland, USA).

#### Gating strategy

Gating strategy for analysis of ex vivo re-stimulated PBMC is shown in Additional file 2: Figure S2. Each gate was set in the negative control sample and then adjusted to the PP and TP stimulated samples with consideration of T-cell receptor downregulation. Two independent audits were performed to control the gating. According to the differential expression of IFN-γ, TNF, and IL-2 the CD4 and CD8 T-cell subpopulations were defined, respectively.

#### Data interpretation

After background subtraction, using the software Pestle version 1.7 (Mario Roederer, ImmunoTechnology Section, VRC/NIAID/NIH, USA), an individual threshold level was calculated for each subpopulation. Values less than zero can occur in cases where the mock-stimulated sample showed more events in a particular functional gate than the antigen-stimulated sample. We applied a threshold according to a previously published method [45]. Positivity thresholds were determined for every CD4 and CD8 T-cell subpopulation using the 90th percentile cut-off based on the negative values after background subtraction. This is important for adequate data correction since unspecific background decreases with the number of positive functions. After threshold application all values lower than the respective individual threshold level were set to zero. Furthermore, a cut-off (assay detection limit) of 0.005% was applied for total CD4 and CD8 T-cell responses. Total CD4 and CD8 T-cell response was calculated by summing up all values of subpopulations, which were positive for at least one of the cytokines IFN-γ, TNF or IL-2 and their respective combinations. Functional composition (cytokine expression pattern) of responding T cells is shown as pie charts, showing the portion of responding T-cell subpopulations of the total antigen-specific response according to their functionality (mono- to tri-functional) upon stimulation, using SPICE software version 5.3 (Mario Roederer, ImmunoTechnology Section, VRC/NIAID/NIH, USA)] [45]. Pie arcs show the cytokines produced by the different subpopulations upon stimulation. Raw data of all performed assays can be provided upon request.

### The IFN-γ ELISpot assay

IFN-γ ELISpot assays (T-Track^®^ human IFN-γ ELISpot kit HiSpecificity^PRO^, Lophius Biosciences, Regensburg, Germany) were performed according to the manufacturer´s instructions. 2 × 10^5^ PBMC/well were plated in a final volume of 150 μL/well and stimulated with TP of EBV-BZLF1, EBV-EBNA3A, HCMV-IE1, and HCMV-pp65, respectively.

#### Data acquisition

ELISpot plates were evaluated within 3 days after assay performance using an automated reader system (CTL-ImmunoSpot^®^ S6 Ultra-V Analyzer/CTL ImmunoSpot 5.4 Professional DC Software, CTL Europe, Bonn, Germany). We followed the guidelines for the automated evaluation of ELISpot assays to account for harmonization efforts for the ELISpot assay [46]. ELISpot plates were scanned with automatically adjusted settings conducted by the reader. Counting of spot forming cells (SFC) within ELISpot plates was performed by adjusting the background balance using the automatic Smart Count™ programme of the CTL software. Counting was performed in compliance with the guidelines for the automated evaluation of ELISpot assays [46] and our laboratory standard counting parameters consisting of a best possible spot separation and a counting mask size of 90%. Spot counts were normalized to 100% of the well area. All obtained counts were reviewed and certified by a second person during a quality control process.

#### Interpretation of results

Final results are represented as IFN-γ spot forming cells (SFC) per 2 × 10^5^ PBMC. Denoted results represent background-subtracted data. The median background reactivity (spot counts in negative control wells) observed within the ELISpot assay was zero spots per well (range 0–3 SFC/well) in IFN-γ ELISpot assays. Positive reactivity to experimental stimulatory agents was selected as a *p*-value of equal or smaller than 0.05 when applying the distribution free sampling method (DFR(2×)), by using a web-based tool (http://www.scharp.org/zoe/runDFR/) [47]. In addition, only mean spot counts of at least 11 SFC/2 × 10^5^ PBMC were regarded as a positive reactivity. Raw data of all performed assays can be provided on reasonable request.

### Statistical analysis

All results were included in the analysis, as no attempt was made to exclude outliers. Concordance with EBV-/HCMV-serology and specificity were defined as the proportion of correctly identified positive and lacking T-cell responses in EBV- and/or HCMV-seropositive and -seronegative individuals, respectively. All tests were two-sided and conducted on exploratory 5% significance levels. Effect measures are presented with 95% confidence intervals. Nonparametric statistical tests were applied in all cases. Paired Wilcoxon signed rank tests were used to assess significance of change in values between experiment conditions. The software Graph Pad Prism 5.00 (GraphPad Software, La Jolla, California, USA) was used for statistical analyses.

### Laboratory environment

Well-trained personnel performed all assays according to SOPs. The same individual throughout the course of the study performed the assays.

## Results

### T-activated EBV- and HCMV-derived proteins assure a high specificity for T-cell assays

First, we examined specificity of T-cell responses against EBV-BZLF1, -EBNA3A, HCMV-IE1, and -pp65 TP in a cohort of EBV- and HCMV-seronegative donors (n = 7), where an absence of a recall response to EBV- and HCMV-derived antigens is expected (Additional file 3: Table S3). Determination of T-cell responses against EBV-EBNA3A TP revealed a specificity of 86% with only 1/7 tested donors showing a low number of antigen-reactive cells (15 IFN-γ SFC/2 × 10^5^ PBMC). No antigen-reactive IFN-γ SFC were detectable after EBV-BZLF1, HCMV-IE1, and HCMV-pp65 TP stimulation in the seronegative control groups, denoting a specificity of 100% of tested TP. To demonstrate the detection of antigen-specific cells within this assay type we additionally determined numbers of EBV- and HCMV-reactive IFN-γ SFC of donors with confirmed positive EBV- and HCMV-serostatus, respectively (Additional file 3: Table S3).

These results proved a high specificity of EBV and HCMV TP-based immune monitoring and indicated these antigens suitable for use in immunological endpoint assays monitoring EBV- and HCMV-specific cellular immune responses in a clinical trial setting.

### T-activated proteins and peptide pools induce similar response rates in ex vivo T-cell assays

Clinical immune monitoring comprises quantification and phenotypic analysis of antigen-reactive T cells to be correlated with the clinical outcome. We compared the response rates to PP and TP stimulation (i.e. their stimulatory capacity) using freshly isolated PBMC of 30 EBV- and 19 HCMV-seropositive individuals.

**Fig. 1:**
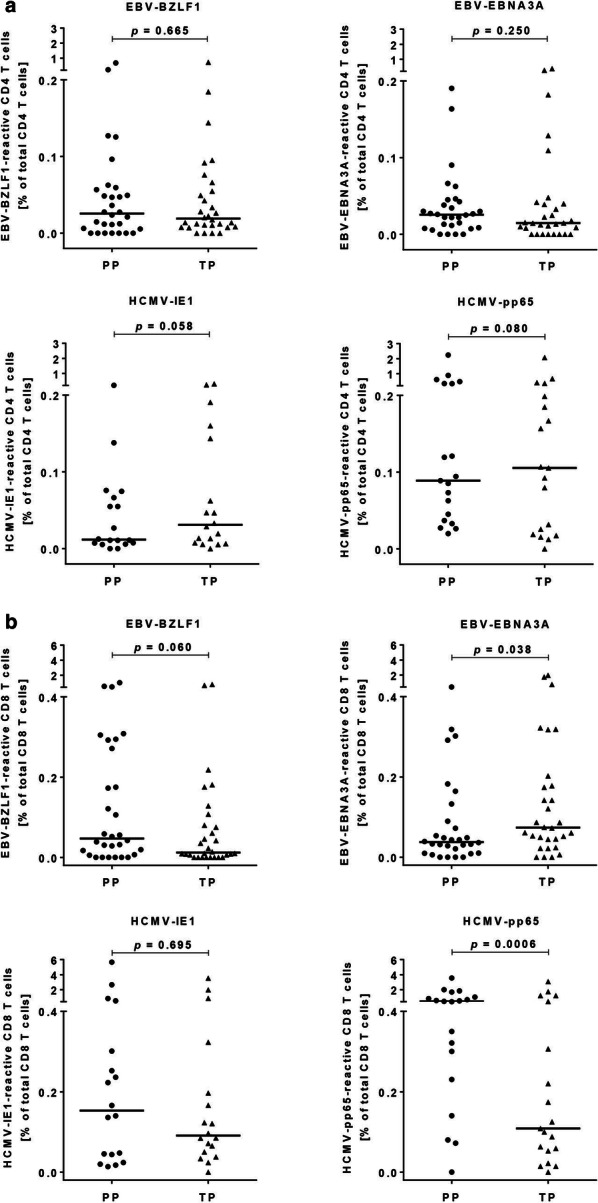
Frequencies of EBV and HCMV T-activated protein and peptide pool-reactive CD4 (**a**) and CD8 (**b**) T cells. Depicted are the frequencies of antigen-reactive CD4 (**a**) and CD8 (**b**) T cells upon stimulation of freshly isolated PBMC with EBV-BZLF1 (n = 30), EBV-EBNA3A (n = 30), HCMV-IE1 (n = 19), and HCMV-pp65 (n = 19) T-activated proteins and the corresponding peptide pools. Dots and triangles represent frequencies of antigen-reactive T cells of single donors after re-stimulation of PBMC with PP (left, dots) and TP (right, triangles), respectively. Frequencies of antigen-reactive T cells were calculated as the sum of IFN-γ, TNF, and IL-2 producing CD4 or CD8 T cells (defined as total response) expressed as a percentage in relation to total CD4 or CD8 T-cell numbers. The horizontal lines indicate the medians. Statistical analyses were done with paired Wilcoxon signed rank tests. *PP* peptide pool, *TP* T-activated protein

The overall magnitude of EBV- and HCMV-reactive CD4 T cells was comparable irrespective of the antigen type. Median frequencies of PP- versus TP-reactive CD4 T cells were 0.026 vs. 0.019% (EBV-BZLF1; *p* = 0.665), 0.026 vs. 0.015% (EBV-EBNA3A; *p* = 0.250), 0.012 vs. 0.031% (HCMV-IE1; *p* = 0.058), and 0.089 vs. 0.106% (HCMV-pp65; *p* = 0.080), respectively (Fig. 1a). However, we observed an intra-individual variation with some subjects showing a higher response rate to one of the two stimulants (PP or TP) (Additional file 4: Figure S4A).

The overall magnitude of PP- versus TP-induced CD8 T-cell responses was similar for EBV-BZLF1 (*p* = 0.060) and HCMV-IE1 (*p* = 0.695), but not for EBV-EBNA3A (*p* = 0.038) and HCMV-pp65 (*p* = 0.0006) (Fig. 1b). We detected median frequencies of PP- versus TP-reactive CD8 T cells of 0.047 vs. 0.012% (EBV-BZLF1), 0.038 vs. 0.074% (EBV-EBNA3A), 0.153 vs. 0.091% (HCMV-IE1), and 0.448 vs. 0.109% (HCMV-pp65), respectively (Fig. 1b). Again we observed striking differences of the individual response rates to PP or TP-derived antigenic stimulation (Additional file 4: Figure S4B).

### T-activated proteins and peptide pools trigger different cytokine expression pattern of EBV- and HCMV-reactive CD8 T cells

Next, we compared the cytokine expression pattern (i.e. the proportion of antigen-reactive cells secreting only one or more cytokines in parallel) of EBV- and HCMV-reactive CD4 and CD8 T cells upon stimulation of freshly isolated PBMC with PP versus TP in 30 EBV- and 19 HCMV-seropositive individuals.

**Fig. 2:**
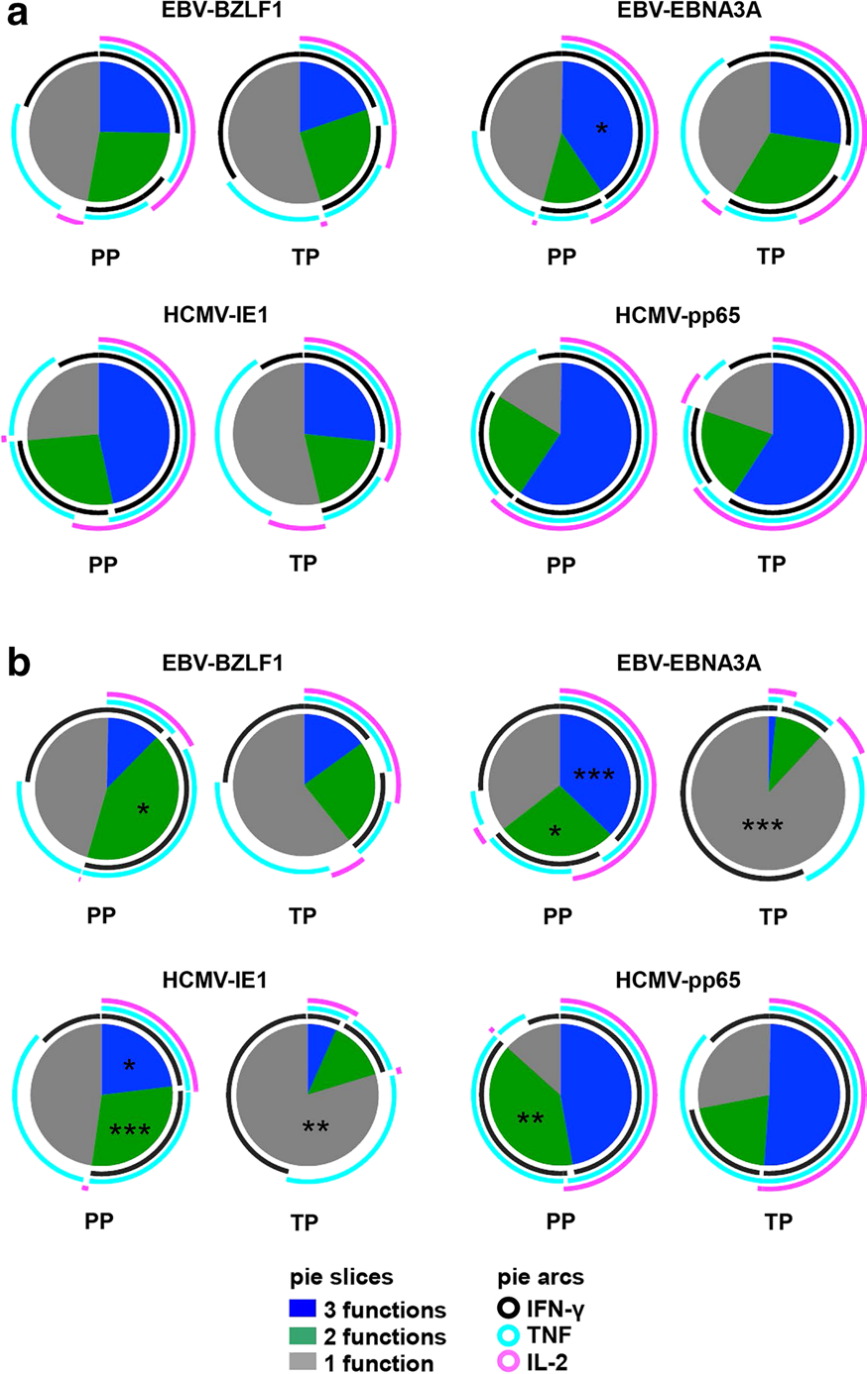
Cytokine expression pattern of EBV- and HCMV-reactive CD4 (**a**) and CD8 (**b**) T cells. Pie charts represent the relative functional composition of total EBV- and HCMV-reactive CD4 (**a**) and CD8 (**b**) T-cell responses upon stimulation of freshly isolated PBMC of 30 EBV- and 19 HCMV-seropositive individuals with T-activated proteins versus the corresponding peptide pools. Each pie slice corresponds to the proportion of T cells positive for one, two or three cytokines (IFN-γ, TNF, and/or IL-2, respectively) (mono-, bi-, and tri-functional T-cell responses, respectively). Individual cytokine expression pattern of mono-, bi-, and tri-functional T-cell responses are illustrated by the concentric colored arcs surrounding the pie chart. Color codes defining the number (slices) and type (arcs) of expressed cytokines are indicated in the figure legend. Statistical analyses of differences of proportions of mono-, bi-, and tri-functional T-cell responses upon stimulation of either PP or TP were done with paired Wilcoxon signed rank tests. *PP* peptide pool, *TP* T-activated protein. *p < 0.05; **p < 0.01; ***p < 0.001

The overall cytokine expression pattern was similar for EBV- and HCMV-reactive CD4 T cells, except for EBV-EBNA3A-reactive T cells (Fig. 2a). For EBV-EBNA3A-reactive T cells we determined a significant higher proportion of tri-functional CD4 T cells (*p* = 0.045) upon stimulation with the PP compared to stimulation with the corresponding TP (Fig. 2a).

Differences in the cytokine expression pattern after PP versus TP stimulation were even more pronounced for the CD8 T-cell responses with the most striking variances for EBV-EBNA3A and HCMV-IE1 reactive CD8 T cells (Fig. 2b). Stimulation with EBV-EBNA3A and HCMV-IE1 PP induced significant higher proportions of bi- (*p* = 0.018 and *p* = 0.0005, respectively) and tri-functional (*p* = 0.0001 and *p* = 0.040, respectively) CD8 T cells, but lower numbers of mono-functional CD8 T cells (*p* < 0.0001 vs. *p* = 0.002) compared to stimulation with EBV-EBNA3A and HCMV-IE1 TP (Fig. 2b). Stimulation with EBV-BZLF1-derived PP revealed significant higher numbers of bi-functional CD8 T cells (*p* = 0.042) compared to the TP-based monitoring, what we also observed for HCMV-pp65 PP-based stimulation (*p* = 0.005) (Fig. 2b).

In summary, ex vivo T-cell stimulation using PP and TP induced almost similar cytokine expression pattern in CD4, but significant distinct cytokine expression pattern in antigen-reactive CD8 T cells.

### Cryopreservation affects T-activated protein- and peptide pool-induced CD8 but not CD4 T-cell reactivity

**Fig. 3:**
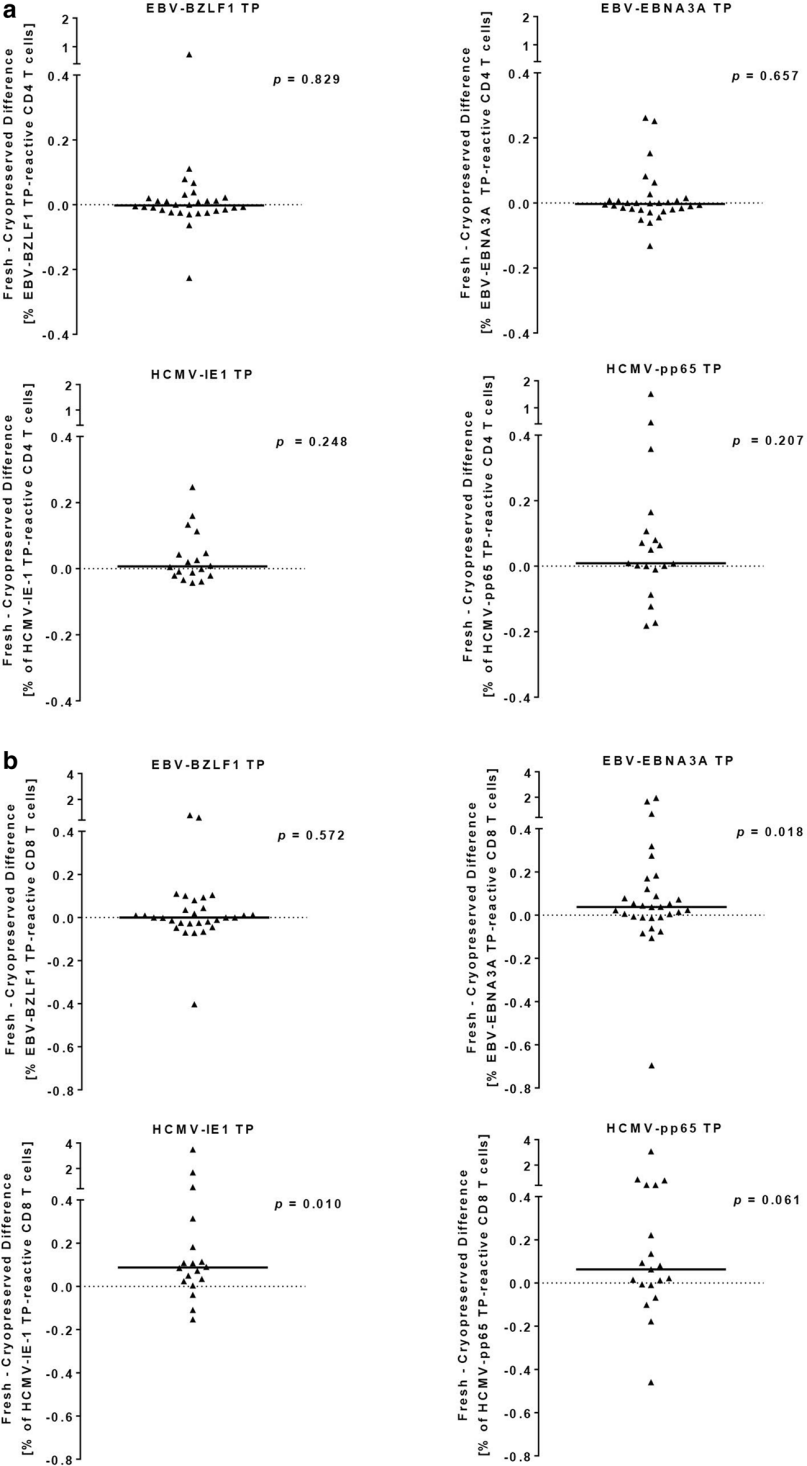
Within-donor differences of T-activated protein reactive CD4 (**a**) and CD8 (**b**) T-cell responses are shown for freshly isolated versus cryopreserved PBMC. Depicted are the within-donor differences of detected CD4 (**a**) and CD8 (**b**) T-cell responses upon stimulation of freshly isolated versus cryopreserved PBMC with T-activated EBV-BZLF1 (n = 30), EBV-EBNA3A (n = 30), HCMV-IE1 (n = 19), and HCMV-pp65 (n = 19) proteins. To determine the bias between freshly isolated and cryopreserved PBMC the frequencies of antigen-reactive CD4 (**a**) or CD8 (**b**) T cells in cryopreserved PBMC was subtracted from that of respective freshly isolated PBMC for each donor. Each triangle represents the respective fresh minus cryopreserved difference of CD4 (**a**) and CD8 (**b**) TP-reactive T-cell responses of each single donor. Bars represent the median difference of antigen-reactive CD4 (**a**) or CD8 (**b**) T-cell responses of all tested donors. All statistics are based on paired Wilcoxon signed rank tests. *PP* peptide pool, *TP* T-activated protein

**Fig. 4:**
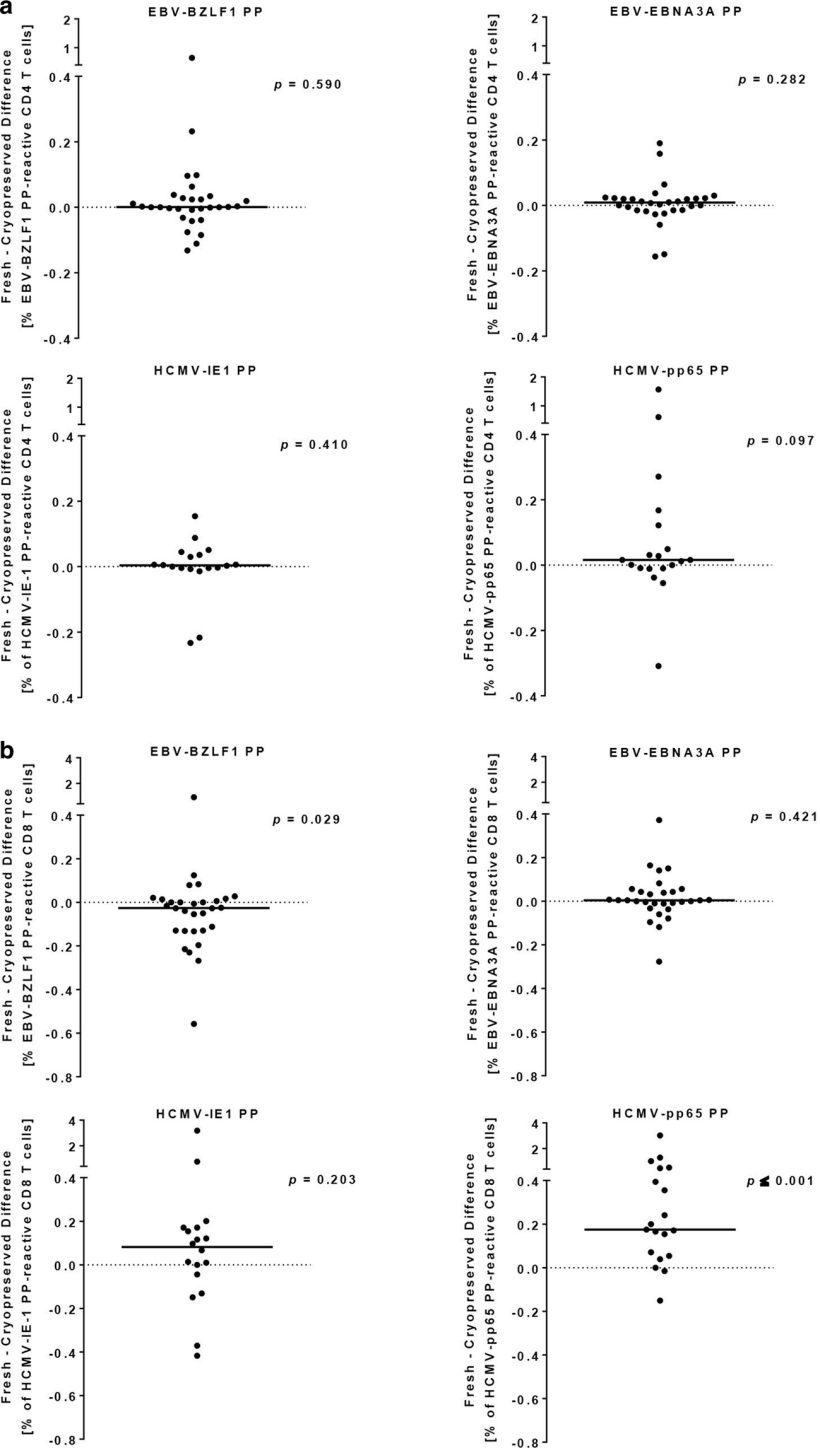
Within-donor differences of peptide pool reactive CD4 (**a**) and CD8 (**b**) T-cell responses are shown for freshly isolated versus cryopreserved PBMC. Depicted are the within-donor differences of detected CD4 (**a**) and CD8 (**b**) T-cell responses upon stimulation of freshly isolated versus cryopreserved PBMC with peptide pools of EBV-BZLF1 (n = 30), EBV-EBNA3A (n = 30), HCMV-IE1 (n = 19), and HCMV-pp65 (n = 19) proteins. To determine the bias between freshly isolated and cryopreserved PBMC the frequencies of antigen-reactive CD4 (**a**) or CD8 (**b**) T cells in cryopreserved PBMC was subtracted from that of respective freshly isolated PBMC for each donor. Each dot represents the respective fresh minus cryopreserved difference of CD4 (**a**) and CD8 (**b**) TP-reactive T-cell responses of each single donor. Bars represent the median difference of antigen-reactive CD4 (**a**) or CD8 (**b**) T-cell responses of all tested donors. All statistics are based on paired Wilcoxon signed rank tests. *PP* peptide pool, *TP* T-activated protein

Clinical immune monitoring often requires the use of cryopreserved PBMC. To determine whether cryopreservation affects the PP- and TP-based monitoring differently, we tested freshly isolated and cryopreserved PBMC samples of 30 EBV- and 19 HCMV-seropositive subjects in each case. Both sample types were stimulated with EBV-BZLF1, EBV-EBNA3A, HCMV-IE1, and HCMV-pp65 TP and PP and the frequencies of antigen-reactive CD4 and CD8 T cells were analyzed by flow cytometry-based ICS (Figs. 3, 4). To determine the bias between freshly isolated and cryopreserved PBMC the frequencies of antigen-reactive CD4 or CD8 T-cells in cryopreserved PBMC was subtracted from that of respective freshly isolated PBMC for each donor.

We did not detect significantly different EBV and HCMV TP-induced CD4 T-cell responses between the two samples types (paired Wilcoxon signed rank tests) (Fig. 3a). The median difference (fresh minus cryopreserved) in TP-reactive CD4 T-cell responses was − 0.003% (− 0.223 to 0.731%), − 0.003% (− 0.132 to 0.262%), 0.007% (− 0.043 to 0.247%), and 0.009% (− 0.182 to 1.515%) for EBV-BZLF1, EBV-EBNA3A, HCMV-IE1, and HCMV-pp65 re-stimulated PBMC, respectively.

By contrast, frequencies of EBV-EBNA3A and HCMV-IE1 TP-reactive CD8 T cells were significantly higher when using PBMC ex vivo compared to cryopreserved specimen (*p* = 0.018 and *p* = 0.010, respectively) (Fig. 3b). In detail, comparison of freshly isolated with cryopreserved PBMC revealed median differences in TP-reactive CD8 T-cell responses of 0% (− 0.403 to 0.778%), 0.038% (− 0.695 to 1.942%), 0.088% (− 0.153 to 3.491%), and 0.063% (− 0.459 to 3.081%) for EBV-BZLF1, EBV-EBNA3A, HCMV-IE1, and HCMV-pp65 re-stimulated PBMC, respectively.

We also did not detect significantly different numbers of EBV and HCMV PP-reactive CD4 T cells between freshly isolated and cryopreserved PBMC (Fig. 4a). Here we detected median differences in PP-reactive CD4 T-cell responses of freshly isolated vs. cryopreserved PBMC of 0.001% (− 0.132 to 0.648%), 0.009% (− 0.156 to 0.190%), 0.004% (− 0.233 to 0.154%), and 0.016% (− 0.309 to 1.566%) for EBV-BZLF1, EBV-EBNA3A, HCMV-IE1, and HCMV-pp65 re-stimulated PBMC, respectively.

However, cryopreservation significantly reduced the number of detectable EBV-BZLF1 and HCMV-pp65 PP-reactive CD8 T cells (*p *= 0.029 and *p* ≤ 0.001, respectively) (Fig. 4b). In detail, comparing freshly isolated with cryopreserved PBMC revealed median differences in PP-reactive CD8 T-cell responses of − 0.026% (− 0.558 to 0.920%), 0.004% (− 0.277 to 0.372%), 0.082% (− 0.417 to 3.178%), and 0.175% (− 0.151 to 3.024%) for EBV-BZLF1, EBV-EBNA3A, HCMV-IE1, and HCMV-pp65 re-stimulated PBMC, respectively.

Based on these results, we calculated the overall response rates of the two sample types to EBV and HCMV TP and PP stimulation (Table 1). Regardless of the type of immunogenic stimulant (TP or PP), the response rate was higher in most settings when PBMC were tested ex vivo. We observed the most striking difference for HCMV-reactive TP-induced CD8 T-cell reactivity in cryopreserved PBMC with a 22% (HCMV-IE1) and 21% (HCMV-pp65) reduction compared to the ex vivo setting (Table 1). Response rates of cryopreserved PBMC to PP stimulation was a maximum of 7% and 10% lower (EBV-BZLF1 and EBV-EBNA3A, respectively) (Table 1). Our data showed that cryopreserved PBMC can be used for a TP-based immune monitoring of EBV- and HCMV-reactive CD4 T cells, and for CD8 T-cell monitoring when taking into account the possible lower response rates.

**Table 1:** Response rates to EBV and HCMV T-activated proteins and peptide pools

Antigen	PBMC specimen	T-cell population	TP-induced reactivity (%)^a^	PP-induced reactivity (%)^a^
EBV-BZLF1	Fresh	CD4	87	77
	Cryo		77	70
	Fresh	CD8	73	80
	Cryo		67	87
EBV-EBNA3A	Fresh	CD4	73	87
	Cryo		87	77
	Fresh	CD8	90	87
	Cryo		80	77
HCMV-IE1	Fresh	CD4	94	89
	Cryo		83	83
	Fresh	CD8	94	100
	Cryo		72	94
HCMV-pp65	Fresh	CD4	95	100
	Cryo		95	100
	Fresh	CD8	95	95
	Cryo		74	100

^a^Reactivity defines the percentage of reactive donors after stimulation of freshly or cryopreserved PBMC of EBV- and HCMV-seropositive donors with the respective antigens

## Discussion

Monitoring of EBV-/HCMV-specific cell-mediated immunity (CMI) is co-decisive for antiviral therapy in transplant settings and is often determined as an immunological endpoint in clinical trials [13, 14, 48]. Assessment of HCMV-specific T-cell responses was reported to be an adequate tool to decide on the appropriate level of immunosuppressive therapy in transplant patients to avoid HCMV-reactivation but also graft rejection [14, 15, 35]. Both approaches require highly sensitive and validated immunoassays, and optimal antigenic stimulants to monitor virus-specific CMI [49].

We hypothesize that T-activated proteins (TP) may be superior to commonly used stimulatory viral antigen preparations (i.e. overlapping peptide pools, PP), because they enable an immune monitoring close to the patients in vivo situation by considering natural antigen processing pathways and activation of all clinically relevant effector cell populations [34]. TP as stimulatory antigens may improve immunological endpoint assays, monitoring virus-specific CMI in a clinical trial setting [35, 36]. In this study, we performed comparative analyses of frequencies, phenotype, and cytokine expression pattern of T cells stimulated by EBV-BZLF1, -EBNA3A, HCMV-IE1, and -pp65 TP or the corresponding overlapping PP. In addition, we investigated the suitability of TP as antigenic component in immunoassays with regard to specificity and stimulatory capacity.

Currently used stimulatory antigens are mainly full-length recombinant proteins or corresponding synthetic PP [50, 51]. Both are useful in addressing CD4 or CD8 related T-cell responses, however, PP-based monitoring may consider certain key functions of CMI (i.e. antigen processing/presentation by APC and the communication between immune cells) insufficiently [16]. The debilitating influence of immunosuppressive drugs on T-cell immunity and APC function has been extensively investigated [28–31]. Using overlapping PP for detection of virus-specific T-cell responses does not necessarily require peptide processing by APC [16]. It is known that extracellular proteases can trim peptides to an optimal length and therefore overcome the natural intracellular processing step of viral proteins by APC [16, 52]. Hence, a diminished function of APC caused by immunosuppressive therapies may not be considered by a PP-based immune monitoring approach. In contrast, exogenous antigens like whole viral proteins and viral TP, need to undergo natural antigen processing pathways and are presented by APC [34, 53].

One of our main findings was that the overall magnitude of ex vivo detectable antigen-reactive CD4 T cells was comparable, irrespective of the recall antigen confirming the usability of TP to monitor frequencies of antigen-reactive CD4 T cells. In contrast, we observed significant higher median frequencies of EBV-EBNA3A, but significant lower median frequencies of HCMV-pp65 reactive CD8 T cells upon TP vs. PP stimulation. The used PP contain 15 mers, which is a suboptimal length for MHC class I restricted epitopes [24, 54], whereas peptides bound by MHC class II typically range from 12 to 20 aa in length [55, 56]. The amino acid length of single peptides, as well as the position of the T-cell epitope within a peptide, can greatly influence results of T-cell based immunoassays [26]. Since extracellular proteases can trim peptides to a more optimal length fitting MHC class I molecules [52], the standard format of 15 mer peptides could represent a good compromise for stimulating both CD8 and CD4 T-cell responses and is widely used for the monitoring of antigen-reactive CD8 T cells. However, it is likely, that peptide trimming by proteases may result in peptides differing in length other than intracellular processing and that these differently generated peptides address various subsets of memory T cells.

TP are cross-presented to CD8 T cells via MHC class I molecules [33]. Thus, immunoassays based on peptides resulting either from natural processing (TP) or trimming (PP) may address and activate different subsets of virus-specific memory T cells. Indeed, at least our results for EBV-EBNA3A and HCMV-pp65 reactive CD8 T cells seem to confirm this hypothesis. This is in line with clinical reports, showing that a certain peptide based ex vivo monitoring of virus-specific T cells did not correlate with the patient´s clinical outcome [32, 57]. EBV-EBNA3A and -BZLF1 TP are currently evaluated as one of three antigenic components in an ongoing, prospective multicentre clinical observational study (Munich infectious mononucleosis (IMMUC) study). The IMMUC study aims at the identification of biomarkers and causative factors of complicated and/or protracted EBV-associated IM. Correlation analysis of detected EBV TP- and PP-triggered T-cell responses with serological and clinical data will provide data on the clinical relevance of a TP-based immune monitoring.

Interestingly, for some individuals we determined a high T-cell response only upon stimulation with one of the two types of antigenic stimulants (either PP or TP), which was more pronounced for the CD8 T-cell subset. Based on the differences of individual TP and PP processing and trimming, we speculate that on a single subject level TP-derived peptides can significantly differ from the corresponding PP composition. For many viral antigens it is yet unknown what proportion of the processed peptides is able to trigger immune responses, because not all peptides presented on MHC class I or II molecules are immunogenic [58]. Profiling of a certain viral immunopeptidome by mass spectrometry to identify MHC ligands and testing of their immunogenicity in vivo, as recently shown for vaccinia virus-derived peptide ligands, would promote the selection of optimal antigenic stimulants [58].

Another important observation of our study was that for all tested EBV- and HCMV-derived antigens, TP and PP trigger significantly different cytokine expression pattern of antigen-reactive CD8 T cells, whereas CD4 T-cell cytokine expression pattern was only different for EBV-EBNA3A reactive CD4 T cells. T-cell cytokine profiling is often determined by immunological endpoint assays monitoring virus-specific T cells in a clinical trial setting to identify correlates of protection [13, 37–39, 59, 60]. There are first hints that a PP-based immune monitoring cannot predict the clinical outcome in certain patient cohorts. Results of a HCMV PP-based CD8 T-cell monitoring of patients under corticosteroid therapy do not correlate with clinical outcome and monitored CD8 T cells do not seem to be protective against viral reactivation [32]. Also La Rosa et al. did not detect any association between HCMV-pp65 and -IE1 PP-triggered T-cell responses and HCMV-associated disease and viremia in liver transplant patients 3 months after engraftment [57]. However, Sester et al. [61] reported of a correlation and predictive potential of HCMV-specific CD4 T cells with virus control and HCMV-associated disease, using HCMV antigen for ex vivo analysis. This is in line with our results showing comparable cytokine expression profiles of HCMV-pp65, -IE-1, and EBV-BZLF1 reactive CD4 T cells upon stimulation with PP and TP.

Testing the clinical validity (as performed in our current IMMUC study for EBV-specific T-cell responses) will investigate whether TP-based T-cell cytokine profiling is superior in reflecting the in vivo situation in patients and is suitable to identify correlates of protection.

The evaluation of antigens as stimulatory components for T-cell monitoring also includes proving for specificity (true negative rate) or presence of cross-reactive T-cell responses [44, 50, 62, 63]. We determined a specificity of 100% for EBV-BZLF1, HCMV-IE1, and -pp65 TP and 86% for EBV-EBNA3A TP. T-cell responses against EBV-EBNA3A detected in one out of seven EBV-seronegative individuals does not match necessarily a false positive response. Savoldo et al. [64] reported of EBV-seronegative subjects who had sporadically detectable EBV DNA loads and EBV-reactive T cells. They assumed that most likely those subjects failed to produce EBV-specific antibodies, but were able to establish EBV-specific memory T cells [64]. Existence of heterologous T cells cross-reactivity between EBV and other antigens has already been described [65, 66] and may explain the detection of EBV-EBNA3A reactive T cells in a single EBV-seronegative subject. To exclude false positive responses reliably, we would recommend determining a positivity cut-off based on data of a sufficiently large cohort of individuals seronegative for the respective pathogen.

Immunological endpoint assays monitoring antigen-reactive T-cell responses can be performed on freshly isolated or cryopreserved specimen. Analysis of fresh PBMC, although logistically challenging, is often preferred because cryopreservation might affect functional and phenotypic properties of cells [67, 68]. Previous reports showed that cryopreservation has no negative influence on the overall percentage of CD4 and CD8 T-cell subsets [67] as well as on certain CD4 T-cell subpopulations, like naïve (CD4+ CD45RA+ CD95−) and activated (CD4+ CD38+ HLA-DR+) CD4 T cells [69]. Other T-cell subpopulations, like activated CD8 T cells (CD8+ CD38+ HLA-DR+) or memory CD4 T cells (CD4+ CD45RO+) were reported to be decreased in cryopreserved compared to fresh PBMC [69]. Contradictory data exist on alterations of antigen-specific T-cell responses upon cryopreservation [67, 70, 71]. However, contradictions might be explained by different sources (healthy individuals vs. patients) and processing procedures (freezing/thawing SOPs) of biosamples.

Besides reported changes in the immunophenotype and alterations in antigen-specific T-cell responses, the usage of cryopreserved PBMC in a central immune monitoring unit minimize inter-assay and inter-laboratory variation both being well-known problems in many multi-centre clinical trials with local sample processing [70, 71].

To evaluate the influence of the sample material we determined the overall response rates to EBV-BZLF1, -EBNA3A, and HCMV-IE1, and -pp65 TP or their corresponding overlapping PP with freshly isolated versus cryopreserved PBMC in parallel.

With freshly isolated PBMC, we observed a comparable high response rate for both TP- and PP-based assay set-ups. Furthermore, the concordance with HCMV-serology of the TP-based assay was in line with previously published data on HCMV-specific antigens generated with freshly isolated PBMC [35, 36, 50, 72]. For both antigenic stimulants, we observed lower response rates when using cryopreserved PBMC. Thus, a proportion of TP-reactive T cells will not be considered when using cryopreserved PBMC, but the same holds true to a lesser extent for a PP-based monitoring. One explanation for the decreased stimulatory capacity of TP on cryopreserved PBMC could be that (cross-) presentation of MHC class I restricted peptides implies a fully functional processing of proteins by APC, which might be affected in cryopreserved material [16, 73]. However, based on our results, we can rule out a generally impaired APC function due to cryopreservation as HCMV-pp65 TP triggered CD4 T-cell responses in 95% of all tested individuals irrespective of whether freshly isolated or cryopreserved PBMC were used.

## Conclusion

Based on our evaluation, we conclude that EBV- and HCMV-derived T-activated proteins are suitable antigenic components for an ex vivo monitoring of antigen-reactive CD4 and CD8 T cells and with restrictions also when using cryopreserved PBMC.

## Supplementary information

**Additional file 1: Table S1:** Antibodies used for ICS.

**Additional file 2: Figure S2.** Representative gating strategy for polychromatic intracellular cytokine staining assay.

**Additional file 3: Table S3:** Specificity of EBV-BZLF1, EBV-EBNA3A, HCMV-pp65, and HCMV-IE1 T-activated proteins in a cohort of EBV- and HCMV-seronegative donors.

**Additional file 4: Figure S4.** Inter-individual variations of frequencies of EBV and HCMV T-activated protein and peptide pool-reactive CD4 (A) and CD8 (B) T cells.

## Data Availability

All data generated or analyzed during this study are included in this published article and its supplementary information files. Raw data of all performed assays can be provided on reasonable request.

## Abbreviations

aa: Amino acid
APC: Antigen presenting cells
CD: Cluster of differentiation
CMI: Cell-mediated immunity
DMSO: Dimethyl sulfoxide
EBNA: Epstein–Barr virus nuclear antigen
EBV: Epstein–Barr virus
ELISA: Enzyme-linked immunosorbent assay
ELISpot: Enzyme-linked immuno spot
FACS: Fluorescence activated cell sorting
FCS: Fetal calf serum
HCMV: Human cytomegalovirus
ICS: Intracellular cytokine staining
IFN: Interferon
IL: Interleukin
IM: Infectious mononucleosis
MIATA: Minimum Information About T-cell Assays
MHC: Major histocompatibility complex
PBMC: Peripheral blood mononuclear cells
PBS: Phosphate buffered saline
PP: Peptide pool
RPMI: Roswell Park Memorial Institute
SFC: Spot forming cells
SOP: Standard operating procedure
TNF: Tumor necrosis factor
TP: T-activated protein
