# Supplementary material for: Evaluation of T-activated proteins as recall antigens to monitor Epstein–Barr virus and human cytomegalovirus-specific T cells in a clinical trial setting
Source: J Transl Med. 2020 Jun 17;18:242. doi: 10.1186/s12967-020-02385-x (PMC7298696; doi:10.1186/s12967-020-02385-x)
Supplement: Supplementary file 1 — Additional file 1: Table S1: Antibodies used for ICS. [file 12967_2020_2385_MOESM1_ESM.pdf]

Additional file 1: Table S1. Antibodies used for ICS

**Antibody**	**Assay concentration (µg/mL)**	**Company**	**Clone**	**Compensation control**
CD3-V500	2.0	BD Biosciences	SP34-2	
CD4-PerCP	0.24	BD Biosciences	SK3	
CD8-ECD	0.16	Beckman Coulter	2ST8.5H7	
IL2-APC	20.0	BD Biosciences	5344.111	CD8-APC
TNFα-eFluor450	0.05	eBioscience	MAb11	CD3-eF450
IFNγ-Al700	0.4	BD Biosciences	B27	CD3-Al700
