# Supplementary material for: Evaluation of T-activated proteins as recall antigens to monitor Epstein–Barr virus and human cytomegalovirus-specific T cells in a clinical trial setting
Source: J Transl Med. 2020 Jun 17;18:242. doi: 10.1186/s12967-020-02385-x (PMC7298696; doi:10.1186/s12967-020-02385-x)
Supplement: Supplementary file 2 — Additional file 2: Figure S2. Representative gating strategy for polychromatic intracellular cytokine staining assay. [file 12967_2020_2385_MOESM2_ESM.pdf]

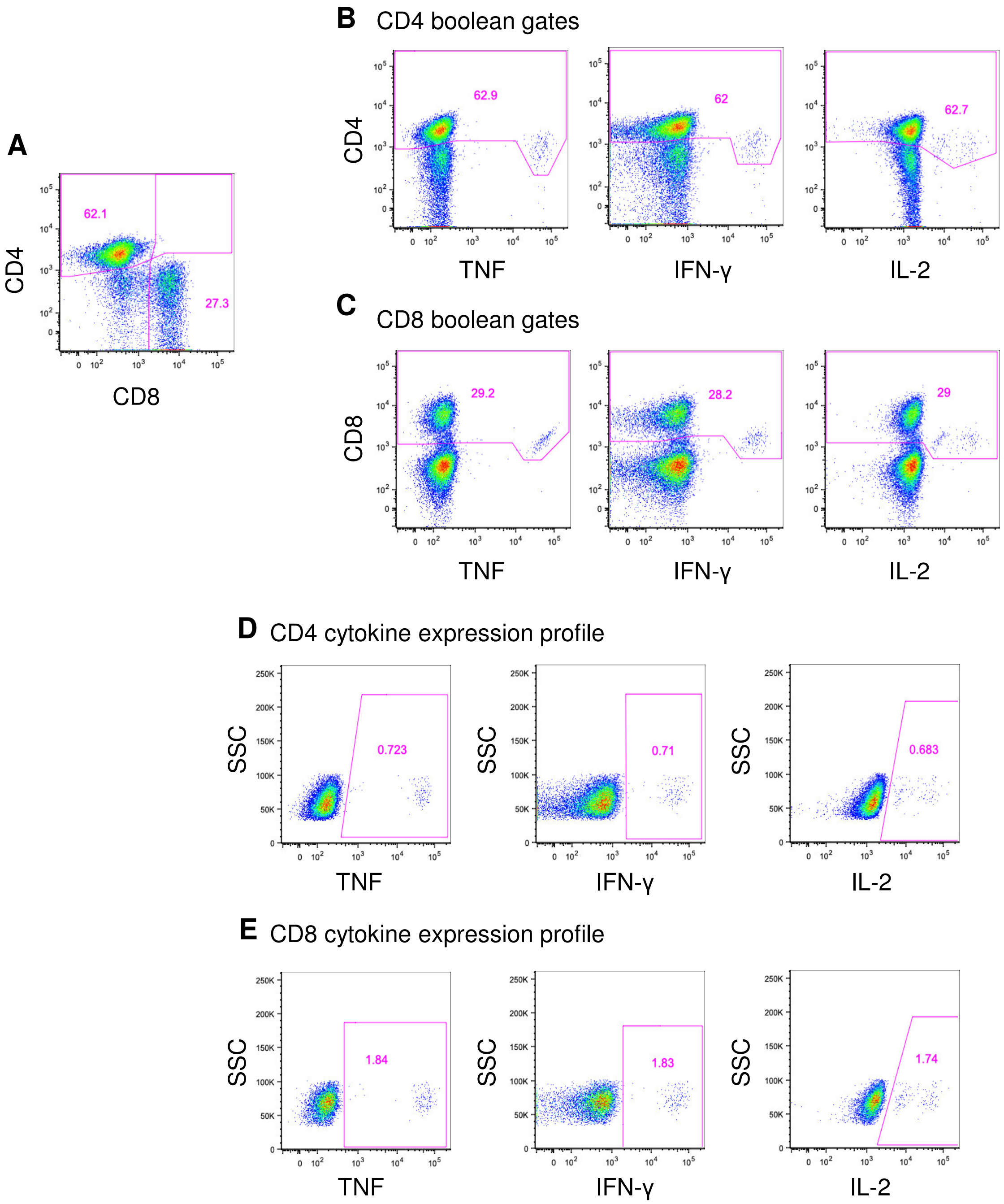

Additional file 2: Figure S2. Representative gating strategy for polychromatic intracellular cytokine staining assay. Lymphocytes were gated based on FSC versus SSC plot, followed by exclusion of dead cells by NIR staining, and gating on CD3 positive cells (data not shown). As representatively shown, CD4 and CD8 cells were gated on all three cytokines to account for downregulation of antigen-specific responding T cells and all these gates were combined with the Boolean operator "OR" to obtain the CD4 and CD8 cell population (B and C, resp.). Thereby, CD4 T cells were excluded from the CD8 T-cell population and vice versa (A). Once CD4 T-cell population was defined, CD4 T cells (D) positive for TNF, IFN-γ, and IL-2 and CD8 T cells (E) positive for TNF, IFN-γ, and IL-2 were separately identified by using different plots in which the axis were chosen to provide the best discrimination between positive and negative events. Selection of positive cells for the three cytokines was done by comparison with a mock-stimulated sample.
