# Supplementary material for: Evaluation of T-activated proteins as recall antigens to monitor Epstein–Barr virus and human cytomegalovirus-specific T cells in a clinical trial setting
Source: J Transl Med. 2020 Jun 17;18:242. doi: 10.1186/s12967-020-02385-x (PMC7298696; doi:10.1186/s12967-020-02385-x)
Supplement: Supplementary file 3 — Additional file 3: Table S3: Specificity of EBV-BZLF1, EBV-EBNA3A, HCMV-pp65, and HCMV-IE1 T-activated proteins in a cohort of EBV- and HCMV-seronegative donors. [file 12967_2020_2385_MOESM3_ESM.pdf]

Additional file 3: Table S3. Specificity of T-activated EBV- and HCMV-proteins

Antigen	Donor	EBV-/CMV-serostatus	SFC[a]/2x10^5 PBMC	Specificity
EBV-BZLF1	S01	negative	0	100 %
EBV-BZLF1	S02	negative	0	
EBV-BZLF1	S03	negative	0	
EBV-BZLF1	S04	negative	0	
EBV-BZLF1	S05	negative	0	
EBV-BZLF1	S06	negative	0	
EBV-BZLF1	S07	negative	0	
EBV-EBNA3A	S01	negative	0	86 %
EBV-EBNA3A	S02	negative	0	
EBV-EBNA3A	S03	negative	0	
EBV-EBNA3A	S04	negative	15	
EBV-EBNA3A	S05	negative	0	
EBV-EBNA3A	S06	negative	0	
EBV-EBNA3A	S07	negative	0	
HCMV-IE1	S01	negative	0	100 %
HCMV-IE1	S02	negative	0	
HCMV-IE1	S03	negative	0	
HCMV-IE1	S04	negative	0	
HCMV-IE1	S05	negative	0	
HCMV-IE1	S06	negative	0	
HCMV-IE1	S07	negative	0	
HCMV-pp65	S01	negative	0	100 %
HCMV-pp65	S02	negative	0	
HCMV-pp65	S03	negative	0	
HCMV-pp65	S04	negative	0	
HCMV-pp65	S05	negative	0	
HCMV-pp65	S06	negative	0	
HCMV-pp65	S07	negative	0	
EBV-BZLF1	S08	positive	29	
EBV-BZLF1	S09	positive	148	
EBV-BZLF1	S10	positive	409	
EBV-EBNA3A	S08	positive	40	
EBV-EBNA3A	S09	positive	126	
EBV-EBNA3A	S10	positive	452	
HCMV-IE1	S11	positive	12	
HCMV-IE1	S12	positive	26	
HCMV-IE1	S13	positive	19	
HCMV-pp65	S11	positive	128	
HCMV-pp65	S12	positive	572	
HCMV-pp65	S13	positive	235	

[a] Values are the mean number of antigen-specific IFN-γ SFC/2x10^5 PBMC (stimulated with 10 µg/ml EBV-BZLF1, 15 µg/ml EBV-EBNA3A, 3 µg/ml HCMV-pp65, and 15.6 µg/ml HCMV-IE1 T-activated proteins, respectively. Results illustrate examination of specificity of T-cell responses against EBV-BZLF1, -EBNA3A, HCMV-IE1, and -pp65 TP in a cohort of EBV- and HCMV-seronegative donors, where an absence of a recall response to EBV- and HCMV-derived antigens is expected. Additionally, numbers of SFC of six donors with confirmed positive EBV- and HCMV-serostatus are listed.
