# Supplementary material for: Evaluation of T-activated proteins as recall antigens to monitor Epstein–Barr virus and human cytomegalovirus-specific T cells in a clinical trial setting
Source: J Transl Med. 2020 Jun 17;18:242. doi: 10.1186/s12967-020-02385-x (PMC7298696; doi:10.1186/s12967-020-02385-x)
Supplement: Supplementary file 4 — Additional file 4: Figure S4. Inter-individual variations of frequencies of EBV and HCMV T-activated protein and peptide pool-reactive CD4 (A) and CD8 (B) T cells. [file 12967_2020_2385_MOESM4_ESM.pdf]

Additional file 4 Figure S4 A

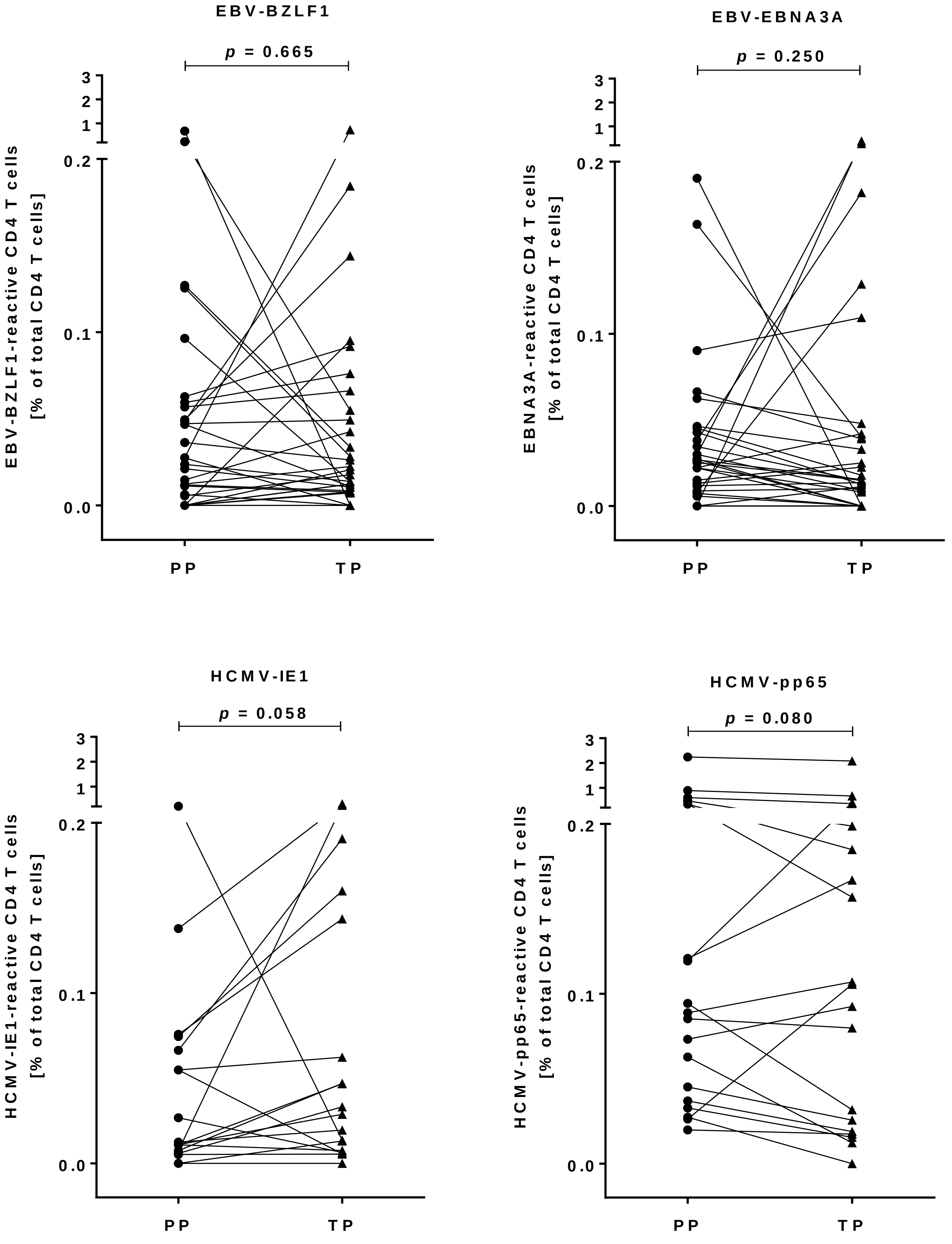

Additional file 4 Figure S4 B

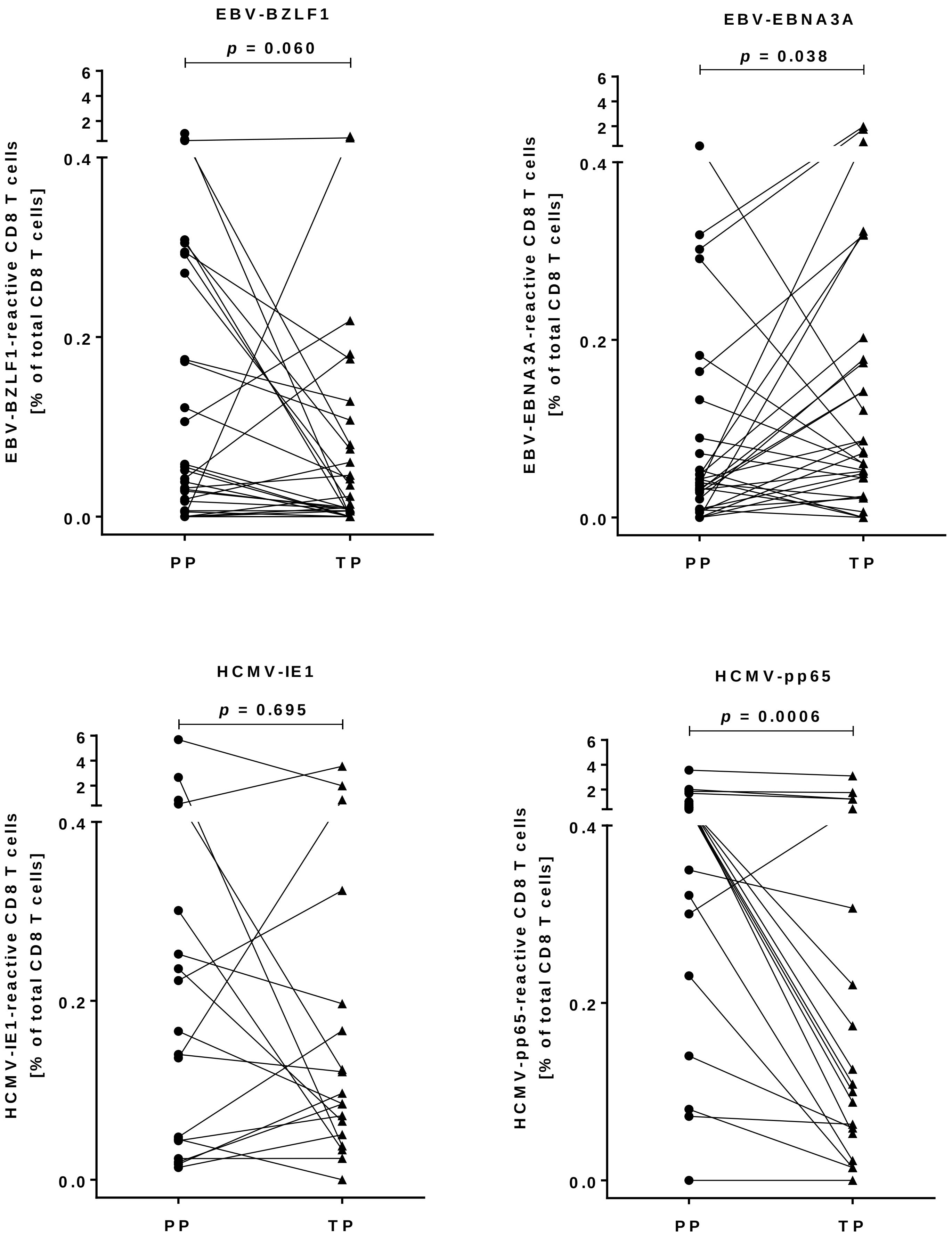

Additional file 4: Figure S4. Inter-individual variations of frequencies of EBV and HCMV T-activated protein and peptide pool-reactive CD4 (A) and CD8 (B) T cells. Depicted are the frequencies of antigen-reactive CD4 (A) and CD8 (B) T cells upon stimulation of freshly isolated PBMC with EBV-BZLF1 (n = 30), EBV-EBNA3A (n = 30), HCMV-IE1 (n = 19), and CMV-pp65 (n = 19) T-activated proteins and the corresponding peptide pools. Dots and triangles represent frequencies of T-cell responses of single donors after re-stimulation of PBMC with PP (left, dots) and TP (right, triangles). Conjugated dots and triangles, representing T-cell responses of single donors, are linked with lines. Frequencies of antigen-reactive T cells are illustrated as the sum of IFN-γ, TNF, and IL-2 producing CD4 or CD8 T cells (defined as total response) expressed as a percentage in relation to total CD4 or CD8 T cells. Statistical analyses were done with paired Wilcoxon signed rank tests. PP = peptide pool; TP = T-activated protein.
